# Disease burden of hepatitis C in the Austrian state of Tyrol – Epidemiological data and model analysis to achieve elimination by 2030

**DOI:** 10.1371/journal.pone.0200750

**Published:** 2018-07-12

**Authors:** Benedikt Schaefer, André Viveiros, Ramona Al-Zoairy, Sarah Blach, Samantha Brandon, Homie Razavi, Livia Dorn, Armin Finkenstedt, Maria Effenberger, Ivo Graziadei, Mario Sarcletti, Herbert Tilg, Heinz Zoller

**Affiliations:** 1 Department of Medicine I, Gastroenterology, Hepatology and Endocrinology, Medical University of Innsbruck, Innsbruck, Austria; 2 Centre for Disease Analysis (CDA), Lafayette, Colorado, United States of America; 3 Department of Medicine II, Sozialmedizinisches Zentrum Ost Donauspital, Vienna, Austria; 4 Department of Medicine, Landeskrankenhaus Hall, Hall/Tirol, Austria; 5 Department of Dermatology and Venereology, Medical University of Innsbruck, Austrian HIV Cohort Study, Innsbruck, Austria; Centers for Disease Control and Prevention, UNITED STATES

## Abstract

**Background:**

In 2016, the World Health Organization (WHO) and 69th World Health Assembly approved the first global health sector strategy (GHSS) on viral hepatitis with the goal to eliminate hepatitis C virus (HCV) infections worldwide. The aim is a 90% reduction of new infections and 65% reduction of HCV-related deaths by 2030.

**Aim:**

This study reports on the epidemiology of HCV infections in the Austrian state of Tyrol (total population 750,000) and uses a predictive model to identify how the WHO strategy for elimination of HCV can be achieved.

**Methods:**

We developed a regional disease burden model based on observed local diagnosis data from 2001 to 2016. Scenarios were developed to evaluate the impact of diagnosis and treatment on HCV-related outcomes (viremic prevalence, decompensated cirrhosis, hepatocellular carcinoma, and liver-related deaths) from 2015 through 2030.

**Results:**

In the last 15 years, 1,721 patients living in Tyrol have been diagnosed with chronic HCV infection. When ageing, mortality and treatment were factored in, there were an estimated 2,043 viremic HCV infections in 2016, of which 1,136 cases had been diagnosed. A baseline model predicts a decrease of 588 HCV cases from 2015 to 2030, which would not translate into the significant reduction of infections needed to achieve WHO global health recommendations. A total of 1,843 infected individuals need to be identified and treated to achieve the WHO goals by 2030 (1,254 averted cases as compared to baseline model). Implementation of this strategy would avoid 523 new HCV infections and decreases HCV-related mortality by 73%.

**Conclusion:**

HCV elimination and >65% reduction of associated mortality are possible for Tyrol, but requires a significant increase in new diagnoses and treatment rate. The model presented in this study could serve as an example for other regions to reliably predict regional disease burden and estimate how WHO goals can be met in the future.

## Introduction

In 2016 the World Health Organization (WHO) released their first global health sector strategy (GHSS) on viral hepatitis[1]. The strategy lays out an ambitious plan to reduce the incidence of global chronic hepatitis infections from the current 6–10 million cases of chronic infection to 0.9 million cases worldwide, and to reduce the annual deaths from chronic hepatitis from 1.4 million to less than 0.5 million by 2030. Approximately 48% of these numbers are attributed to chronic hepatitis C infections. According to the WHO, their goals can be achieved by a reduction of new infections by at least 30% by 2020 and 90% by 2030. A reduction in HCV-related deaths of 10% and 65%, by 2020 and 2030 respectively, is also targeted[1].

In order to establish strategies towards elimination of chronic hepatitis C and achieve the WHO Targets, it is crucial to understand the epidemiology of the disease specific to each country and region. With stronger knowledge of HCV, policy makers can make better decisions about implementing prevention programs as well as the necessary treatment and diagnosis increases needed to achieve the GHSS goals. Current literature estimates the prevalence of anti-HCV infections in Austria to be 0.46% or 37,900 cases in 2008, while viremic prevalence is estimated to be 0.34% (28,000) cases in the same year.[2] However, these estimates have been reported in high risk or unrepresentative populations, such as blood donors[3].

In 2017, Austria took the first step towards HCV elimination by lifting treatment restrictions to allow ≥F0 patients to receive treatment. With free access to DAA therapy and a relatively low prevalence, the ability to cure and eliminate HCV is possible in Austria. Implementation of strategies to achieve these goals requires accurate, regional epidemiological data as a basis for reliable projections of the future disease burden. However, regional estimates of HCV do not exist in the country. In this study, we aimed to first quantify the disease burden of the Austrian state of Tyrol and then develop scenarios to achieve the GHSS goals. We used established, mathematical models to predict the HCV disease burden and interventions needed to achieve the WHO elimination goals, which could also present an example for implementation in other regions with comparable socio-economic status and health systems[4].

## Methods

**Fig 1:**
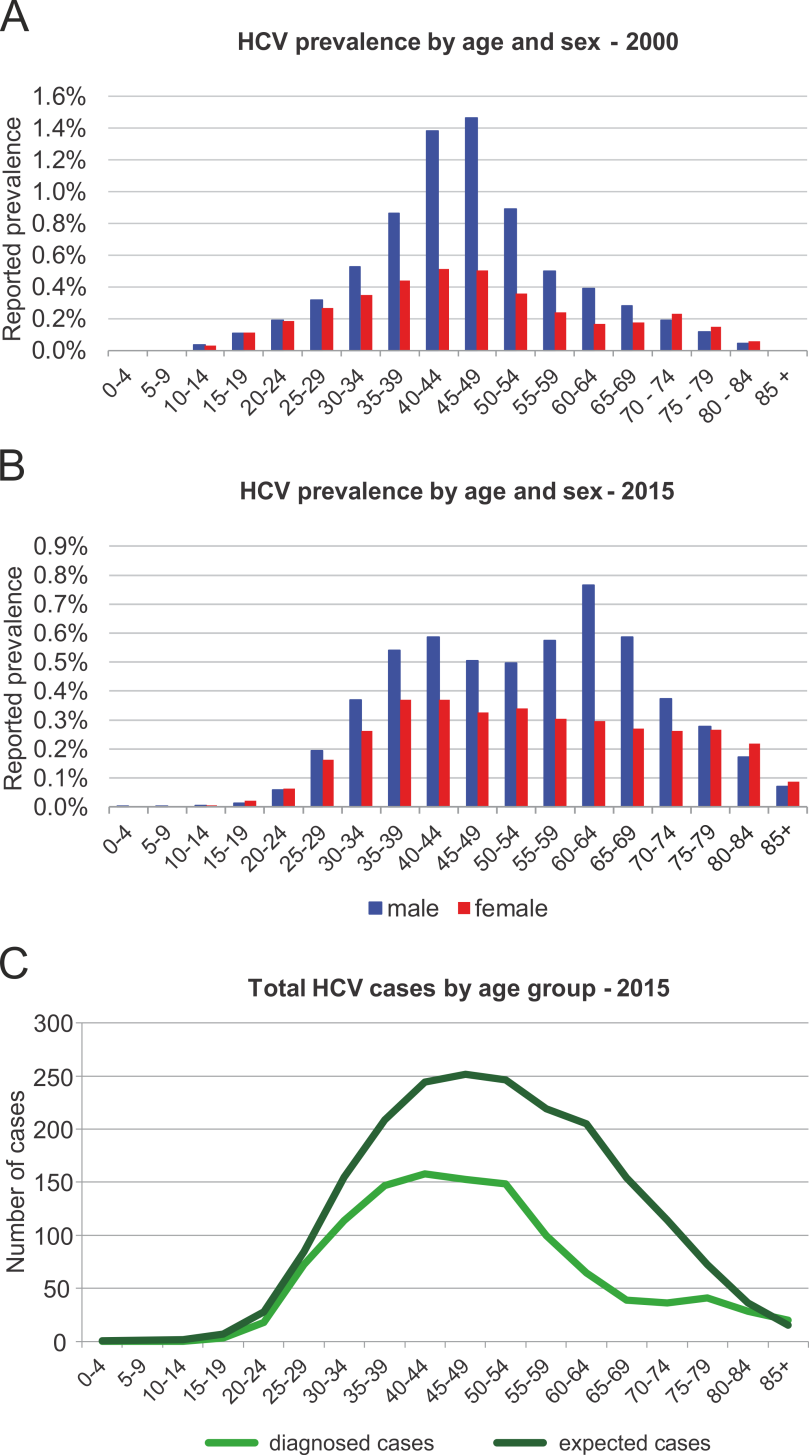
Age and gender distribution of chronic HCV infections in Tyrol for **(A)** 2000 and **(B)** 2015 based on previously reported data.[2] Prevalence estimates for 2015 are based on diagnosed cases and were adjusted for deaths and cures. **(C)** Comparison of expected and observed total HCV cases grouped by age in Tyrol.

The Austrian state of Tyrol, with a population of 746,100 (in 2016), was the focus of this analysis[5]. Epidemiological data was obtained from the Medical University of Innsbruck, and expert estimates were made when data was unavailable. The chronic HCV prevalence in Tyrol was estimated to be the same as national reported prevalence in Austria of 0.34% in 2008 [4]. This estimate is based on information from blood donors, legally mandated notifications to the Federal Ministry of Health and hospital discharge data[2, 3]. However, a reporting bias has been demonstrated in the available epidemiological data and blood donor screening likely underreports HCV prevalence for persons who inject drugs[3]. The model presented in this study factors in this discrepancy in observed and expected cases. So far, local registers of specialized centres are the most reliable source for data on HCV incidence and prevalence in Austria[6]. The age and gender distribution of HCV prevalence was also assumed to mirror that of Austria and is show in Fig 1A. These estimates are based on previously reported epidemiological and demographic information (S1 Appendix).

Since the University Hospital of Innsbruck is the only provider of HCV genotyping in the Austrian state of Tyrol, all patients with suspected HCV infection are referred to this centre for diagnosis. Genotyping was carried out using a validated reversed hybridization assay (Versant HCV Genotype 2.0 LiPA, Siemens, Vienna, Austria). Between January 1^st^ 2001 and December 31^st^ 2016, 1,721 total patients were diagnosed as HCV RNA+ and then were genotyped by the Laboratory of Hepatology, Medical University of Innsbruck. Only minor fluctuations in GT distribution were observed over the last 15 years. GT 1 had the highest prevalence (56.9–67.2%) followed by GT 3 (18.8–37.9%) (Table 1). This hospital data was also used to estimate the total number diagnosed cases in Tyrol. The 1,721 diagnosed HCV RNA+ cases were reported by year, age and gender, and were aged through the model to account for mortality and cures. This resulted in a total of 1,138 living diagnosed cases of chornic HCV in 2016. The HCV prevalence was modelled based on the reported 2008 anti-HCV prevalence estimate of 0.49% (95% CI 0.07; 0.53) and then taking into account the adjusted newly diagnosed chronic HCV infections between 2001 and 2016 (Fig 1B).

**Table 1:** Observed incidence and genotype distribution of HCV infections in Tyrol.

	2001	2002	2003	2004	2005	2006	2007	2008	2009	2010	2011	2012	2013	2014	2015	2016	Total
**GT 1**	129	98	118	82	67	62	57	52	68	41	55	33	40	42	43	42	1029
	60.00%	63.20%	64.80%	56.90%	55.80%	64.60%	57.60%	47.70%	61.30%	58.60%	63.20%	56.90%	58.00%	63.60%	67.20%	55.30%	59.80%
**GT 2**	7	6	9	7	8	5	3	10	9	2	2	2	1	4	4	5	84
	3.30%	3.90%	4.90%	4.90%	6.70%	5.20%	3.00%	9.20%	8.10%	2.90%	2.30%	3.40%	1.40%	6.10%	6.30%	6.60%	4.90%
**GT 3**	72	47	53	51	42	26	35	40	28	23	26	22	25	16	12	25	543
	33.50%	30.30%	29.10%	35.40%	35.00%	27.10%	35.40%	36.70%	25.20%	32.90%	29.90%	37.90%	36.20%	24.20%	18.80%	32.90%	31.60%
**GT 4**	7	4	2	4	3	3	4	7	6	4	4	1	3	4	5	4	65
	3.30%	2.60%	1.10%	2.80%	2.50%	3.10%	4.00%	6.40%	5.40%	5.70%	4.60%	1.70%	4.30%	6.10%	7.80%	5.30%	3.80%
**Total**	215	155	182	144	120	96	99	109	111	70	87	58	69	66	64	76	1721

Treatment is exclusively available through the centre or the connected State Hospital Hall. This allows for more realistic estimates about the incidence of HCV infections and associated complications for this region and enables more precise predictions than previous models.[7, 8]. From January 2014 to December 2016, a total of 404 Tyrolean patients with chronic hepatitis C were treated with interferon-free regimens (S1 Table). Of these 14 (3%) patients relapsed, of whom 12 achieved sustained virological response after a re-treatment. The centre also carries out all liver transplantations done in the region and tracks those patients that received HCV related transplants. Individuals not living in Tyrol were excluded from this study. This retrospective study was carried out with approval of the local ethics research committee (Mitteilungsblatt der Medizinischen Universitaet Innsbruck, June 3rd 2009) and in accordance with national law (Datenschutzgesetz 2000 § 46 Abs. 5).

### Statistical analysis

HCV test results and demographic data were incorporated into a Microsoft Excel based disease progression model, as previously described[7–12]. Briefly, the Markov model was calibrated to historical data and used to forecast the current and future incidence, prevalence of HCV infection and associated disease sequelae[8]. The main input variables of interest included viremic HCV prevalence, genotype distribution, diagnosed, and treated patients (see S1 Appendix). When clinical data were not available, published literature and expert consensus were considered. A Delphi process was used to achieve consensus among authors around model inputs and outputs.

### Model scenarios

Scenarios were developed to evaluate the impact of interventions (diagnosis and treatment) on HCV-related outcomes (viremic prevalence, decompensated cirrhosis, hepatocellular carcinoma, and liver-related deaths) from 2015 through 2030. Two primary scenarios were considered– 1) the current treatment paradigm, or “baseline”; and 2) the level of intervention necessary to eliminate HCV as a public health threat, as defined by the GHSS targets (WHO 2030).

Under the baseline scenario, it was assumed that the both number of treated patients and newly diagnosed patients followed a trend of gradual decline. Ninety-five patients were treated and 70 patients were diagnosed in 2017 decreasing to 47 treated patients and 40 diagnosed patients by 2026. Treatment was restricted to patients between the ages of 20 and 74, but open to all fibrotic stages (≥F0). SVR rates went from 95% in 2017 to 98% in 2018 and remained constant.

Under the GHSS targets (WHO 2030) scenario, assumptions for this model use current diagnosis and treatment rates from the baseline scenario and include the minimal gradual increases in diagnosis and treatment rates required to achieve GHSS targets by year 2030 were estimated. Treatment would need to increase to 130 patients annually by 2019 and could decrease to 120 patients annually by 2026. Diagnosis would need to increase to 120 patients annually by 2018. By 2021, treatment should be available to all patients aged 20–19 and all fibrotic stages starting 2017. SVR rates are held constant at 98% starting in 2018.

## Results

### HCV prevalence

In 2016, the model estimates a total of 2,043 chronic infections in Tyrol. Based on registry data, it was estimated that 46% or 1,136 of the total cases have already been diagnosed (Fig 1C).

### Comparison of modelled scenario outcomes

**Fig 2:**
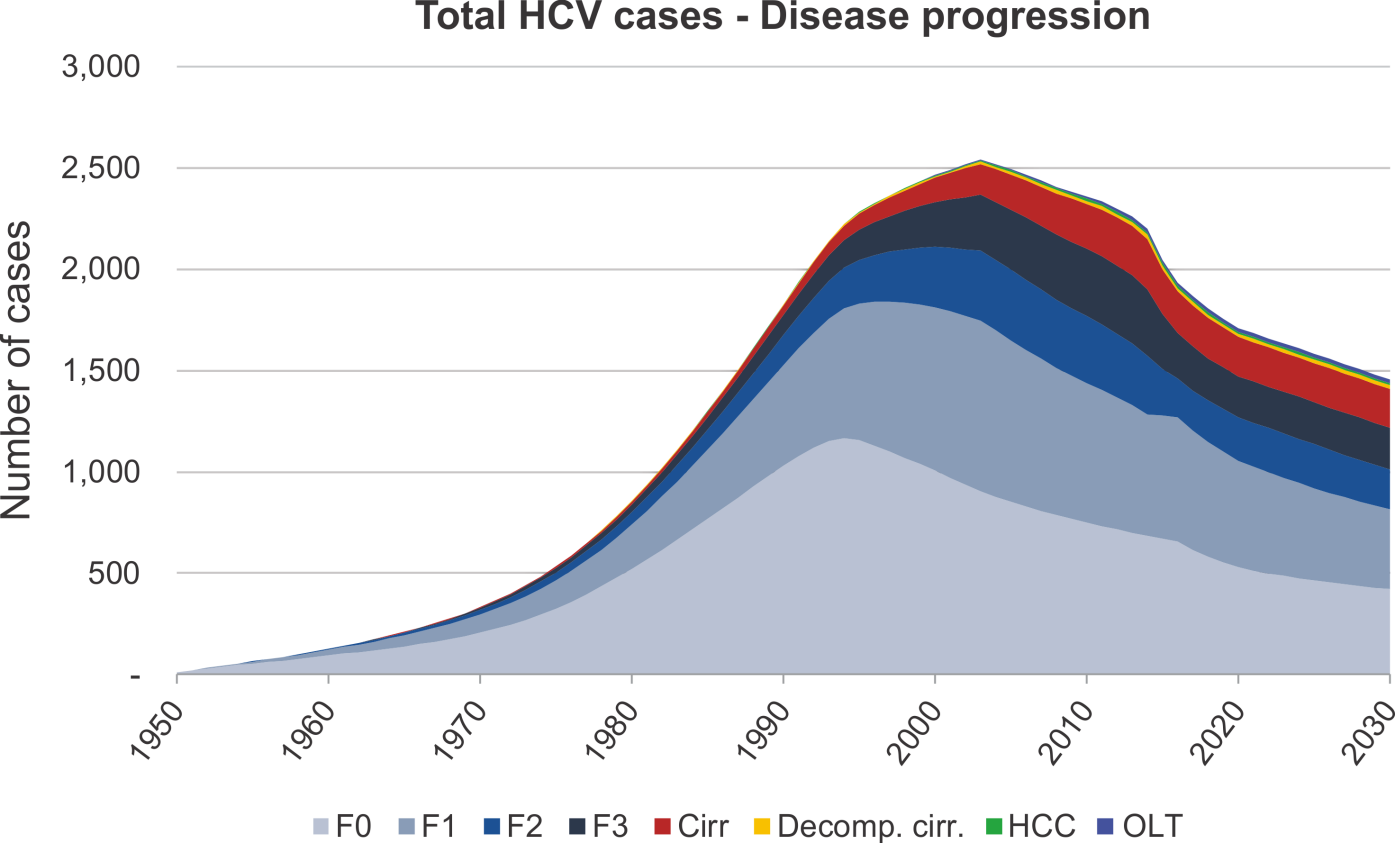
Baseline scenario: Modelled disease progression over time grouped by disease stage. Fibrosis stage 0–3: F0-F3; Cirr: cirrhosis; Decomp. cirr.: decompensated cirrhosis; HCC: hepatocellular carcinoma; OLT: orthotopic liver transplantation.

**Fig 3:**
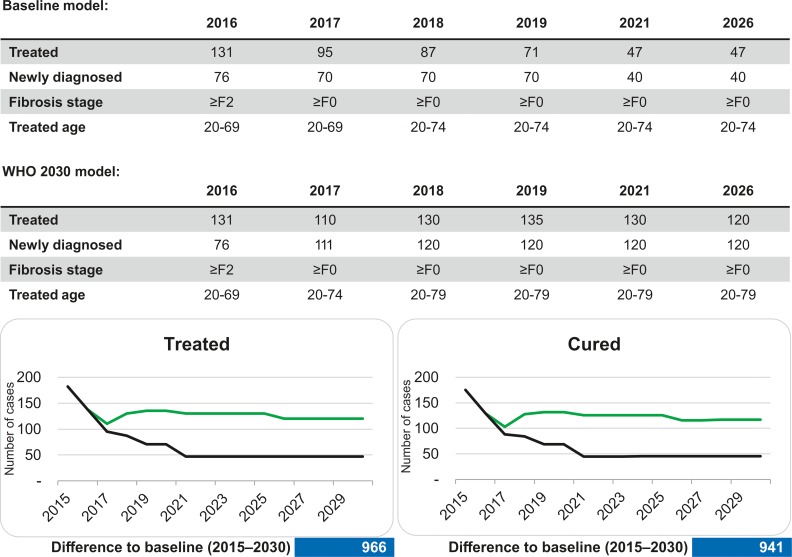
Comparison of baseline model and calculated numbers needed to achieve WHO global health strategies goals by 2030. Black line indicates the baseline scenario, the green line indicates the WHO 2030 model.

Based on the historical local prevalence, incidence and treatment data two scenarios were generated. The first one corresponds to a baseline scenario where the number of patients available for treatment will gradually decrease due to an expected decline in patients available for diagnosis with current public health strategies (Figs 2 and 3).

Since 2003, a steady decline in the number of total HCV cases has been observed. If no changes in treatment and diagnosis are made, the baseline scenario estimates a decrease of 588 HCV cases from 2015 to 2030. This would result in a failure to achieve the WHO global health strategies by 47% (Fig 2).

**Fig 4:**
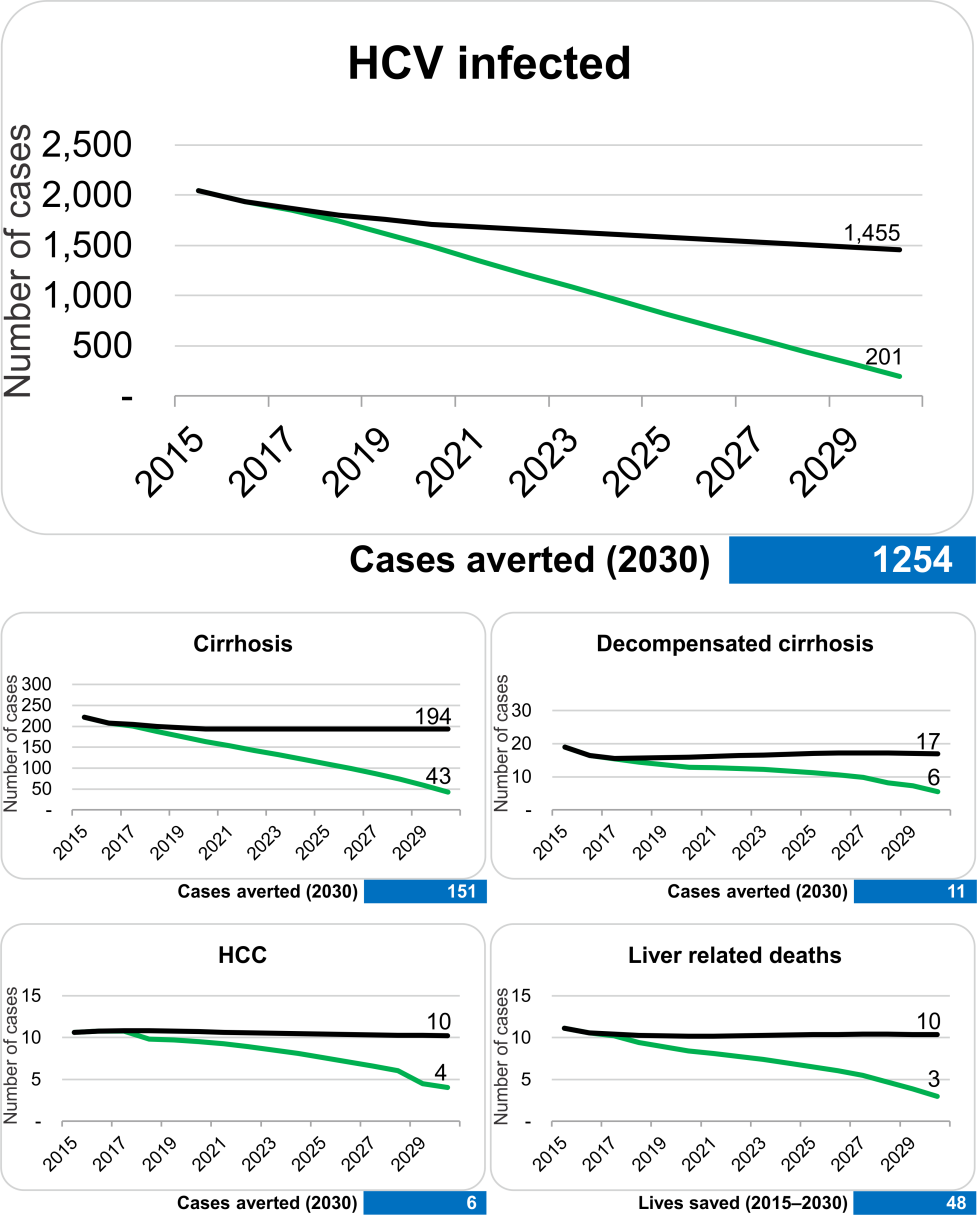
Comparison between total numbers of HCV infections and associated complications in the baseline scenario (black line) and the WHO 2030 model (green line).

**Fig 5:**
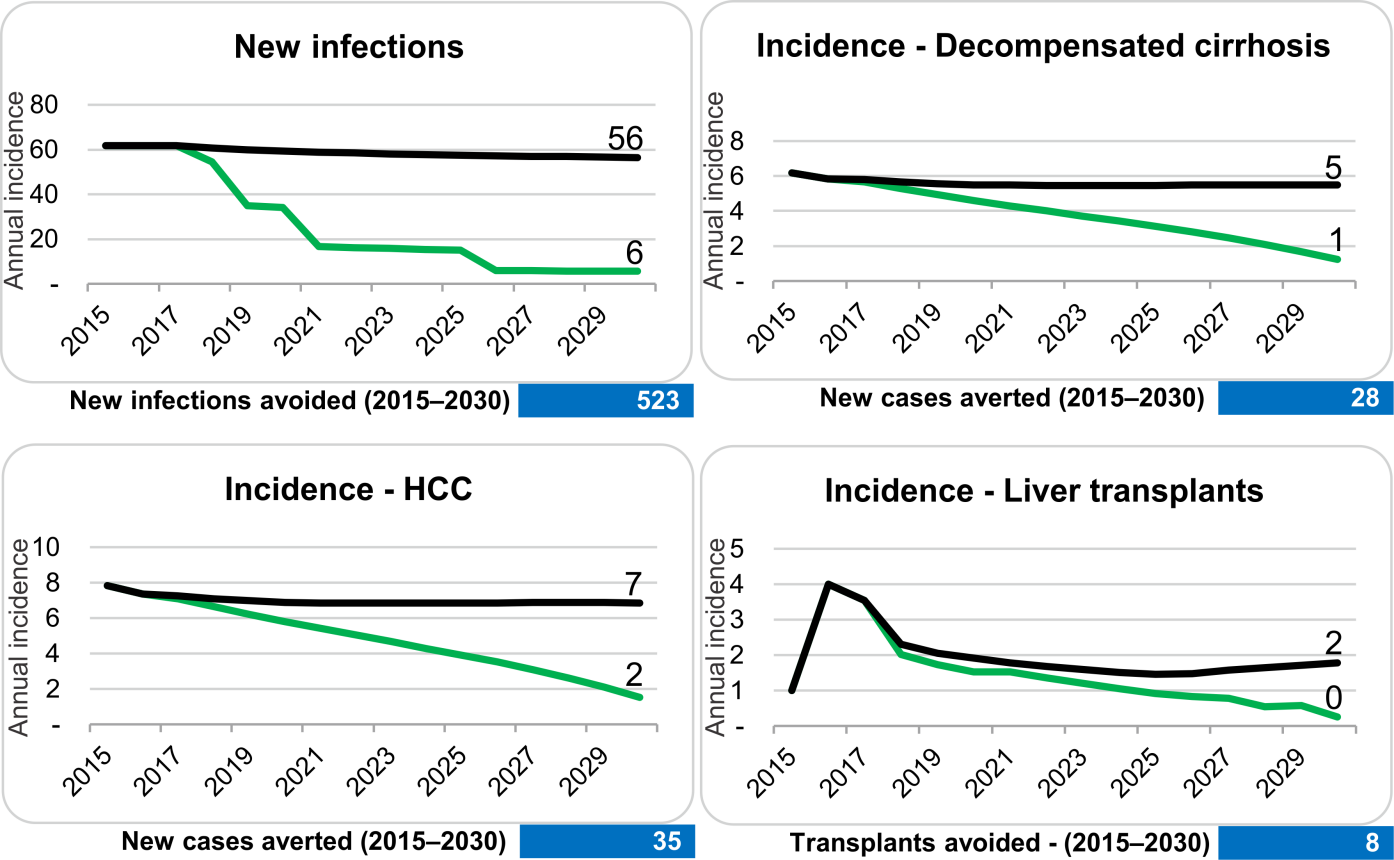
Comparison of incident HCV infections and associated clinical endpoints between baseline (black line) and WHO 2030 models (green line) for Tyrol. Numbers in the blue box indicate the total numbers of averted cases.

The number of new diagnoses, treatments, and reduction in new infections needed to meet the WHO 2030 elimination goals are calculated in the second scenario–the WHO 2030 scenario. To achieve the WHO 2030 elimination targets, an increase in new diagnoses to 120 and treatments to 135 patients yearly by 2019 would be necessary (Fig 3). An impact on clinical endpoints can only be achieved with increased effort in diagnosis, treatment and reduction in new infections. The total number of chronic HCV infections would decrease by 1,843 (90%) individuals between 2015 and 2030, which are 1,254 more averted or cured cases than expected from the baseline model. This would result in a decrease of HCV related mortality by 73% (Fig 4), which corresponds to a total of 48 lives saved. The associated reduction in the total number of HCV related HCC cases would be 35 (62% reduction), 28 for decompensated cirrhosis cases (reduction by 71%) and 8 for HCV related liver transplantations (reduction by 75%, Fig 5). The number of averted new HCV infections would be 573, which corresponds to an incidence reduction of 91% as compared to baseline model (Fig 5).

## Discussion

This analysis presents detailed epidemiological information on HCV prevalence, associated liver disease and strategies to eliminate HCV infections in the Austrian state of Tyrol. In contrast to other regions in Austria, tertiary healthcare, including diagnosis and treatment of HCV, is centralized in the state of Tyrol. This offers a unique opportunity to reliably model HCV disease burden and make future projections for the region. One critical parameter for disease modelling is prevalence of HCV viremia, for which only limited epidemiological data exists in Austria [3, 6, 13, 14].

Our study demonstrates that a significant reduction of liver-related complications and mortality as proposed by the WHO recommendations is a realistic goal. An important step towards achieving HCV elimination is access to treatment for all patients independent of fibrosis stage, which recently has been implemented in Tyrol. Translating the results of this study to increasing the number of new diagnoses will be effective but also the most challenging task. To accomplish a 90% reduction of new infections by 2030 we need to increase diagnoses to more than 120 per year and also treat two-times more HCV infected individuals annually (Fig 3). Even with unlimited and unrestricted access to antiviral HCV therapy, interventions to improve case finding are required[15]. Implementing HCV screening in the high-risk population of people who inject drugs is probably the most cost-effective [16, 17]. The United States Center for Disease Control and Prevention recommends one-time testing for persons born from 1945–1965[18]. For this high-risk birth cohort in Austria, and in particular in Tyrol, screening colonoscopy is a potential screening opportunity, which has been proven to be acceptable and effective[19, 20]. According to the European Association for the Study of the Liver (EASL) and the American Association for the Study of Liver Diseases (AASLD), HCV testing should be performed annually in individuals on opiate substitution therapy and in persons with ongoing risk factors (persons who inject drugs and HIV-infected men who have unprotected sex with men)[21, 22]. Oral fluid anti-HCV antibody point-of-care testing could offer a simple tool to preselect patients with poor venous access. In Tyrol, where the care for patients with HCV is highly centralized but prescription of opiate substitution therapy is decentralized, pre-screening for HCV in this high-risk population with salivary HCV antibody tests could be particularly effective and avoid unnecessary blood sampling.

The forecasts presented in this study could be impacted by a number of limitations. The prevalence estimates are based on national blood donor data, and these types of estimates are not able to account for underreporting in higher risk groups (i.e. injection drug user). To date, there has been no national general population prevalence study, nor has there been any regional study of the kind. Another limitation of this study is that rates and routes of HCV transmission are also based on estimates. As it is unlikely that data on exact prevalence and HCV transmission from representative cohorts reflecting the general Tyrolean population will ever become available from prospective studies, epidemiological modelling remains the most effective and powerful tool to predict the effect of certain scenarios on incidence and prevalence of chronic hepatitis C its sequelae.

Increased HCV awareness, education on modes of transmission as well as information on new therapeutic opportunities offered by novel direct-acting antivirals are needed among primary health care professionals and the general population. This study could guide public health decision making not only to eliminate HCV within the next 15 years, but also to reduce the risk of new HCV infections by reducing HCV prevalence.

## Supporting information

S1 Appendix Detailed description of used methods. (DOCX)

S1 Table Number of Tyrolean patients treated with direct-acting antivirals since 2014. (DOCX)

## Abbreviations

HCV: Hepatitis C
DAAs: direct antiviral agents
GT: genotype
WHO: World Health Organization
GHSS: Global Health Sector Strategy
