# Supplementary material for: Disease burden of hepatitis C in the Austrian state of Tyrol – Epidemiological data and model analysis to achieve elimination by 2030
Source: PLoS One. 2018 Jul 12;13(7):e0200750. doi: 10.1371/journal.pone.0200750 (PMC6042769; doi:10.1371/journal.pone.0200750)
Supplement: S1 Appendix — (DOCX) [file pone.0200750.s001.docx]

**S1 Appendix**

Contents

[Text A: General population assumptions 2](#_Toc510594676)

[Fig. A: The flow of the HCV disease progression model 3](#_Toc510594677)

[Fig. B: Tornado diagram highlighting the key drivers for the 2016 viremic prevalence estimate 4](#_Toc510594678)

[Table A: HCV disease progression rates 5](#_Toc510594679)

[Table B: Parameters included in sensitivity and uncertainty analysis 6](#_Toc510594680)

[Table C: Data sources used for epidemiological models 6](#_Toc510594681)

# Text A: General population assumptions

*Tyrol Region General Population*

Population data were obtained through the Federal Institute of Statistics Austria (FIS) and the United Nations (UN) Population Database (1;2). As data through FIS were only available in 10 to 15-year intervals from 1880-2011, calculations were necessary to adjust the UN data to match local data. Population estimates were calculated by taking a ratio of the population in the Tyrol region relative to Austria. The ratio was used to scale down the country of Austria’s population to reflect just the Tyrol region.

*Tyrol General Mortality Rates*

Mortality rates were obtained through the Max Planck Institute for Demographic Research’s Human Mortality Database (HMD) (3). Mortality data was available for the country of Austria, we assumed that the same rates applied to the Tyrol region

# Fig. A: The flow of the HCV disease progression model

Source: The Polaris Observatory HCV Collaborators. Global prevalence and genotype distribution of hepatitis C virus infection in 2015: a modelling study. Lancet 2016; 2(3): 161-76.

A disease progression model (5,15) was constructed in Microsoft Excel^®^ (Microsoft Corp., Redmond, WA) to quantify the size of the viremic HCV infected population, by the liver disease stages (METAVIR score F0-F4), from 1950-2030. The model started with the annual number of acute infections that progressed to chronic HCV infection after accounting for spontaneous clearance of the virus. The progression of these new cases was followed along with all chronic infections from prior years. Disease progression by age and sex was simulated by multiplying the total number of cases at a particular stage of the disease by a progression rate to the next stage. Progression rates are presented in Table S1. Unless specified, the scope of the model was limited to HCV viremic (ribonucleic acid (RNA) positive) cases. Non-HCV-viremic cases (those who spontaneously cleared the virus or were treated and cured) were not considered even though they would test positive to HCV antibodies and may still progress to more advanced stages of liver disease despite viral clearance (6). The total number of cases, at each stage of the disease, was tracked by age and gender. Single-year age cohorts were used through age 84; those aged 85 and older were treated as one cohort. Each year, the population in each age group, except for 85 and older, was moved to the next age cohort to simulate aging.

# Fig. B: Tornado diagram highlighting the key drivers for the 2016 viremic prevalence estimate


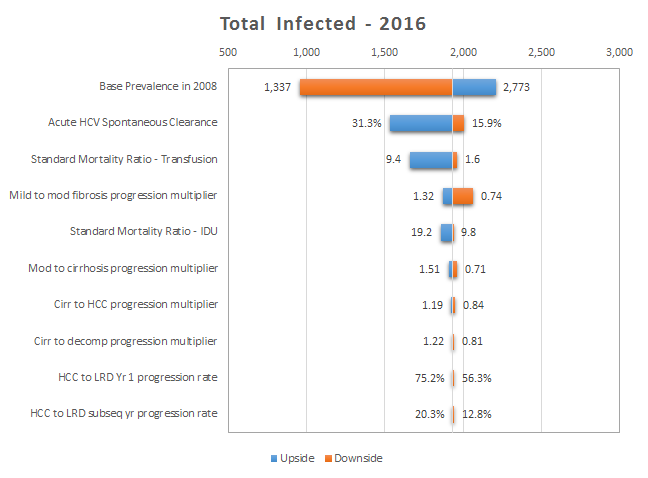


# Table A: HCV disease progression rates

*Reported Progression rates:*

| **Disease Progression** | **Reported Progression Rates** | **Source** |
| --- | --- | --- |
| Acute HCV Spontaneous Clearance | 18.0% (15.0-45.0%) | (7-9) |
| HCC to Liver Related Death (Yr 1) | 70.7% (43-77.0%) | (14;16) |
| HCC to Liver Related Death (Sub Yrs) | 16.2% (11-23.0%) | (16) |
| Liver Transplant to Liver Related Death (Yr 1) | 33.1-10.7% (SD 2.8-0.4%) | (17;18) |
| Liver Transplant to Liver Related Death (Sub Yrs) | 3.9-4.8% (SD 7.6%-1.0) | (17;18) |

Source: Razavi H, Waked I, Sarrazin C, et al. The present and future disease burden of hepatitis C virus (HCV) infection with today's treatment paradigm. J Viral Hepat 2014; 21 Suppl 1: 34-59.

*Progression rates used in the model, back-calculated and adjusted by age and gender:*

| **Age Cohorts** | 0-4 | 5-9 | 10-14 | 15-19 | 20-24 | 25-29 | 30-34 | 35-39 | 40-44 | 45-49 | 50-54 | 55-59 | 60-64 | 65-69 | 70-74 | 75-79 | 80-84 | 85+ |
| --- | --- | --- | --- | --- | --- | --- | --- | --- | --- | --- | --- | --- | --- | --- | --- | --- | --- | --- |
| **Back-calculated progression rates - males** | | | | | | | | | | | | | | | | | | |
| F0 to F1 | 5.3% | 5.3% | 6.4% | 6.4% | 5.2% | 5.2% | 3.8% | 3.8% | 13.9% | 13.9% | 17.1% | 17.1% | 19.4% | 19.4% | 21.8% | 21.8% | 17.9% | 17.9% |
| F1 to F2 | 3.8% | 3.8% | 4.7% | 4.7% | 3.8% | 3.8% | 2.7% | 2.7% | 10.1% | 10.1% | 12.4% | 12.4% | 14.1% | 14.1% | 15.8% | 15.8% | 13.0% | 13.0% |
| F2 to F3 | 5.4% | 5.4% | 6.6% | 6.6% | 5.3% | 5.3% | 3.9% | 3.9% | 14.3% | 14.3% | 17.5% | 17.5% | 19.9% | 19.9% | 22.4% | 22.4% | 18.3% | 18.3% |
| F3 to Cirrhosis | 0.0% | 0.0% | 0.8% | 0.8% | 2.5% | 2.5% | 5.7% | 5.7% | 8.8% | 8.8% | 4.8% | 4.8% | 9.9% | 9.9% | 19.1% | 19.1% | 19.1% | 19.1% |
| F3 to HCC | 0.0% | 0.0% | 0.0% | 0.0% | 0.0% | 0.0% | 0.0% | 0.0% | 0.1% | 0.1% | 0.1% | 0.1% | 0.2% | 0.2% | 0.3% | 0.3% | 0.3% | 0.3% |
| Cirrhosis to  HCC | 0.3% | 0.3% | 0.3% | 0.3% | 0.3% | 0.3% | 0.5% | 0.5% | 0.9% | 0.9% | 1.4% | 1.4% | 2.4% | 2.4% | 3.9% | 3.9% | 3.9% | 3.9% |
| **Back-calculated progression rates - females** | | | | | | | | | | | | | | | | | | |
| F0 to F1 | 4.4% | 4.4% | 5.4% | 5.4% | 4.3% | 4.3% | 3.1% | 3.1% | 11.6% | 11.6% | 14.3% | 14.3% | 16.2% | 16.2% | 18.2% | 18.2% | 14.9% | 14.9% |
| F1 to F2 | 3.2% | 3.2% | 3.9% | 3.9% | 3.1% | 3.1% | 2.3% | 2.3% | 8.4% | 8.4% | 10.4% | 10.4% | 11.7% | 11.7% | 13.2% | 13.2% | 10.8% | 10.8% |
| F2 to F3 | 4.5% | 4.5% | 5.5% | 5.5% | 4.4% | 4.4% | 3.2% | 3.2% | 11.9% | 11.9% | 14.6% | 14.6% | 16.6% | 16.6% | 18.6% | 18.6% | 15.3% | 15.3% |
| F3 to Cirrhosis | 0.0% | 0.0% | 0.6% | 0.6% | 2.1% | 2.1% | 4.7% | 4.7% | 7.4% | 7.4% | 4.0% | 4.0% | 8.3% | 8.3% | 15.9% | 15.9% | 15.9% | 15.9% |
| F3 to HCC | 0.0% | 0.0% | 0.0% | 0.0% | 0.0% | 0.0% | 0.0% | 0.0% | 0.0% | 0.0% | 0.1% | 0.1% | 0.1% | 0.1% | 0.2% | 0.2% | 0.2% | 0.2% |
| Cirrhosis to  HCC | 0.3% | 0.3% | 0.3% | 0.3% | 0.3% | 0.3% | 0.4% | 0.4% | 0.7% | 0.7% | 1.2% | 1.2% | 2.0% | 2.0% | 3.3% | 3.3% | 3.3% | 3.3% |

Source: Razavi H, Waked I, Sarrazin C, et al. The present and future disease burden of hepatitis C virus (HCV) infection with today's treatment paradigm. J Viral Hepat 2014; 21 Suppl 1: 34-59

# Table B: Parameters included in sensitivity and uncertainty analysis

| **Parameter** | **Base** | **Low** | **High** | **Source** | **Distribution** |
| --- | --- | --- | --- | --- | --- |
| ***Disease Progression Rates*** |  |  |  |  |  |
| Acute HCV Spontaneous Clearance | 18.0% | 15.0% | 45.0% | (7-9) | Beta-PERT |
| Mild to moderate fibrosis^1^ | 1.00 | 0.59 | 1.53 | *(21) | Triangular |
| Moderate fibrosis to cirrhosis^1^ | 1.00 | 0.57 | 1.90 | *(21) | Triangular |
| Compensated to decompensated cirrhosis^1^ | 1.00 | 0.70 | 1.36 | *(21) | Triangular |
| Cirrhosis to HCC^1^ | 1.00 | 0.74 | 1.32 | *(21) | Triangular |
| HCC to Liver Rel. Death (Yr 1) | 70.7% | 43.0% | 77.0% | (14;16) | Beta-PERT |
| HCC to Liver R. Death (Sub Yrs) | 16.2% | 11.0% | 23.0% | (16) | Beta-PERT |
| Liver Transplant 1988-2010 Rate | 2.2% | 2.1% | 3.2% | Calculated | Beta-PERT |
| Liver Transplant 2011-2030 Rate | 3.0% | 2.9% | 4.5% | Calculated | Beta-PERT |
| ***Input variables*** |  |  |  |  |  |
| Anti-HCV Prevalence Rate - 2008 | 0.46% | 0.07% | 0.53% | (19) | Beta-PERT |
| Standard Mortality Ratio - IDU | 10.0 | 9.5 | 29.9 | (22-24) | Beta-PERT |
| Standard Mortality Ratio - Transfusion | 2.1 | 1.3 | 17.6 | (25) | Beta-PERT |
| ^1^Multipliers are applied to base transition probabilities, by age and gender, during uncertainty analysis  ^2^Cost inputs for uncertainty analysis are presented prior to adjusting for diagnosis rate  ^*^Derived from progression rates published by Harris et al 2013 (28) | | | | | |

# Table C: Data sources used for epidemiological models

| Input | Value | Year | Data source |
| --- | --- | --- | --- |
| Anti-HCV prevalence | 0.46% | 2008 | (19) |
| Viremic rate | 73.9% | 2008 | (19) |
| Genotype distribution | 59.8% GT1; 4.9% GT2; 31.6% GT3 3.8% GT4 | 2008-2016 | Laboratory of Hepatology, Medical University of Innsbruck |
| Cumulatively diagnosed (viremic) | 1,136 | 2001-2015 |  |
| Cumulative liver transplants | 28 | 2001-2016 |  |

**Reference List**

(1) Austrian Federal Institute of Statistics. Register Based Census 2011, population since 1869. 2011 [cited:Mar 01 2017] Available from: URL: http://www.statistik.at/web_en/statistics/PeopleSociety/population/population_censuses_register_based_census_register_based_labour_market_statistics/totaL_population/028544.html

(2) United Nations.Dept.of Economic and Social Affairs.Population Division (2011). World population prospects: The 2010 revision, Volume I: Comprehensive tables. ST/ESA/SER.A/313.2011 Available from: URL: <http://esa.un.org/wpp/Documentation/pdf/WPP2010_Volume-I_Comprehensive-Tables.pdf>

(3) University of California,B, Mack Planck Institute for Demographic Research. Human Mortality Database.Jun 14 2013 [cited:Feb 1 2013] Available from: URL: [www.mortality.org](http://www.mortality.org)

(5) Razavi H, Waked I, Sarrazin C, et al. The present and future disease burden of hepatitis C virus (HCV) infection with today's treatment paradigm. J Viral Hepat 2014; 21 Suppl 1: 34-59.

(6) Aleman S, Rahbin N, Weiland O, et al. A risk for hepatocellular carcinoma persists long-term after sustained virologic response in patients with hepatitis C-associated liver cirrhosis. Clin Infect Dis 2013; 57: 230-6.

(7) Thomas DL, Seeff LB. Natural history of hepatitis C. Clin Liver Dis 2005; 9: 383-98.

(8) Alter MJ, Margolis HS, Krawczynski K, et al. The natural history of community-acquired hepatitis C in the United States. The Sentinel Counties Chronic non-A, non-B Hepatitis Study Team. N Engl J Med 1992; 327: 1899-905.

(9) Villano SA, Vlahov D, Nelson KE, Cohn S, Thomas DL. Persistence of viremia and the importance of long-term follow-up after acute hepatitis C infection. Hepatology 1999; 29: 908-14.

(10) Razavi H, Elkhoury AC, Elbasha E, et al. Chronic hepatitis C virus (HCV) disease burden and cost in the United States. Hepatology 2013; 57: 2164-70.

(11) Deuffic-Burban S, Deltenre P, Buti M, et al. Predicted effects of treatment for HCV infection vary among European countries. Gastroenterology 2012; 143: 974-85.

(12) Thein HH, Yi Q, Dore GJ, Krahn MD. Estimation of stage-specific fibrosis progression rates in chronic hepatitis C virus infection: A meta-analysis and meta-regression. Hepatology 2008; 48: 418-31.

(13) Bennett WG, Inoue Y, Beck JR, Wong JB, Pauker SG, Davis GL. Estimates of the cost-effectiveness of a single course of interferon-alpha 2b in patients with histologically mild chronic hepatitis C. Ann Intern Med 1997; 127: 855-65.

(14) Bernfort L, Sennfalt K, Reichard O. Cost-effectiveness of peginterferon alfa-2b in combination with ribavirin as initial treatment for chronic hepatitis C in Sweden. Scand J Infect Dis 2006; 38: 497-505.

(15) The Polaris Observatory HCV Collaborators. Global prevalence and genotype distribution of hepatitis C virus infection in 2015: a modelling study. Lancet 2016; 2(3): 161-76.

(16) Ries, LAG, Young, GL, Keel, GE, Eisner, MP, Lin, YD, and Horner, MJ. SEER survival monograph: Cancer survival among adults: U.S. SEER program, 1988-2001, patient and tumor characteristics National Cancer Institute, SEER Program; 2007. NIH Pub. No. 07-6215.

(17) Organ Procurement and Transplantation Network (OPTN). National Data. Health Resources and Services Administration, U.S. Department of Health & Human Services; 2013.

(18) Organ Procurement and Transplantation Network (OPTN). 2009 OPTN/SRTR annual report 1999-2008: Table 9.15a. Unadjusted patient survival by year of transplant at 3 months, 1 year, 3 years, 5 years and 10 years, deceased donor liver transplants. 2009 Annual report of the U S Organ Procurement and Transplantation Network and the Scientific Registry of Transplant Recipients: Transplant data 1999-2008 2009 [cited:Oct 19 2012] Available from: URL: <http://www.ustransplant.org/annual_reports/current/915a_li.htm>

(19) Hope VD, Eramova I, Capurro D, Donoghoe MC. Prevalence and estimation of hepatitis B and C infections in the WHO European Region: a review of data focusing on the countries outside the European Union and the European Free Trade Association. Epidemiol. Infect. 2013 May 29; 1-17.

(20) IMS Health. IMS Health MIDAS Data. IMS Health Jan 1 2013 Available from: URL: <http://www.imshealth.com/portal/site/ims/menuitem.edb2b81823f67dab41d84b903208c22a/?vgnextoid=4475e3de7e390310VgnVCM1000007f8c2ca2RCRD>

(21) Harris R, Thomas B, Griffiths Jet al. Provision of care for patients with hepatitis C in England: Future burden of HCV-related liver disease and impact of treatment under different scenarios. J Hepatol. Forthcoming 2013.

(22) Hickman M, Hope V, Coleman B, et al. Assessing IDU prevalence and health consequences (HCV, overdose and drug-related mortality) in a primary care trust: implications for public health action. J Public Health (Oxf) 2009; 31: 374-82.

(23) Degenhardt L, Hall W, Warner-Smith M. Using cohort studies to estimate mortality among injecting drug users that is not attributable to AIDS. Sex Transm Infect 2006; 82 Suppl 3: iii56-iii63.

(24) Mathers BM, Degenhardt L, Bucello C, Lemon J, Wiessing L, Hickman M. Mortality among people who inject drugs: a systematic review and meta-analysis. Bull World Health Organ 2013; 91: 102-23.

(25) Kamper-Jorgensen M, Ahlgren M, Rostgaard K, et al. Survival after blood transfusion. Transfusion 2008; 48: 2577-84.
