# Supplementary material for: Disease burden of hepatitis C in the Austrian state of Tyrol – Epidemiological data and model analysis to achieve elimination by 2030
Source: PLoS One. 2018 Jul 12;13(7):e0200750. doi: 10.1371/journal.pone.0200750 (PMC6042769; doi:10.1371/journal.pone.0200750)
Supplement: S1 Table — (DOCX) [file pone.0200750.s002.docx]

**S1 Table:** Number of Tyrolean patients treated with interferon-free regimens since 2014.

|  | *HCV monoinfected* | *HCV+HIV coinfected* | *Total* |
| --- | --- | --- | --- |
| 2014 | 87 | 9 | 96 |
| 2015 | 152 | 25 | 177 |
| 2016 | 111 | 20 | 131 |
